# Stigma in coronavirus disease-19 survivors in Kashmir, India: A cross-sectional exploratory study

**DOI:** 10.1371/journal.pone.0240152

**Published:** 2020-11-30

**Authors:** Shabir Ahmad Dar, Syed Quibtiya Khurshid, Zaid Ahmad Wani, Aaliya Khanam, Inaamul Haq, Naveed Nazir Shah, Mir Shahnawaz, Hena Mustafa

**Affiliations:** 1 Department of Psychiatry, Government Medical College, Srinagar, India; 2 Department of Surgery, SKIMS Medical College, Srinagar, India; 3 Department of Social and Preventive Medicine, Government Medical College, Srinagar, India; 4 Department of Chest Medicine, Government Medical College, Srinagar, India; Imam Abdulrahman Bin Faisal University, SAUDI ARABIA

## Abstract

**Background:**

Coronavirus disease-19 (COVID-19) has not only spawned a lot of stigma and discrimination towards its survivors but also to their corpses. We aimed to assess the magnitude and correlates of stigma in these survivors, on return to their communities.

**Methods:**

This was a cross-sectional, hospital-based, exploratory study conducted by the postgraduate department of psychiatry, in collaboration with the postgraduate department of chest medicine, Govt. medical college, Srinagar. The study was performed among COVID-19 survivors, who attended the outpatient department after their discharge from the hospital. Socio-demographic characteristics were recorded through semi-structured proforma. Stigma was measured by the stigma questionnaire. Data was analyzed using descriptive statistics and regression analysis.

**Results:**

A total of 91 survivors consented to participate in the study. Almost half (46.2%) of them were in the age group of 30–49 years and close to two-thirds (68.1%) were males. About three–fourths (74.7%) were from the urban background. The mean time from hospital discharge to study entry was 11.7±5.1 [Range(R) = 7–21] days. 98% of survivors provided at least one stigma endorsing response and the total mean stigma score was 28.5±7.1[R = 6–39]. The mean stigma sub-scores were highest for enacted stigma (7.6±1.8) [R = 2–9] and externalized stigma (15.0±4.1) [R = 1–20]. Enacted stigma was significantly high in males as compared to females. Enacted stigma and internalized stigma were both associated with education. Enacted stigma, externalized stigma, disclosure concerns, and total stigma was significantly associated with the occupation. Being unemployed and time since discharge were identified as independent predictors of total stigma.

**Conclusion:**

Our study results showed high levels of enacted and externalized stigma among COVID-19 survivors. Enacted stigma was more among males and in those who were highly educated. Survivor centered and community-driven anti-stigma programs are the need of the hour to promote the recovery and community re-integration of these survivors.

## Introduction

The unprecedented outbreak of the COVID-19 in early December 2019 from a wet market in Wuhan aroused enormous attention globally [1]. As of August 21, 2020, the total number of COVID-19 cases in India are 2,975,701 with 697,330 active cases, and 2,222,577 cured/discharged patients after its first case in the country on January 30, 2020. As many as 55,794 people have died and the recovery rate in the country stands at 74.69 percent [2].

Since the contagion gets rapidly transmitted among the persons in close contact, a wide fragment of the world's population is currently restricted to their homes, owing to nationwide lockdowns and home-confinement strategies [3]. Infected patients may develop severe and even fatal acute respiratory distress syndrome or acute respiratory failure landing up in intensive care [3, 4]. Apart from causing anxiety, depression, posttraumatic symptoms, and grief in the families due to the horrific death of COVID-19 patients, this rapidly spreading and the unpredictable pandemic has lead to discrimination and stigmatization of its survivors [5]. Upon their reintegration in society, the survivors are greeted with hostile stares even from their family members and neighbors [6].

Stigma constitutes negative attitudes and beliefs that discredit an individual or group of individuals leading to prejudice, societal exclusion, discrimination, marginalization, and racism [7]. Thus stigma can lead to experiences and feelings of blame, shame, worthlessness, isolation, social exclusion, and discrimination in accessing social amenities and healthcare services [8, 9]. Socially undesirable manifestations like prejudice and discrimination expressed against those with the stigmatizing attributes are known as enacted stigma whereas the feeling of shame, guilt, or worthlessness experienced as a result of having the stigmatizing attribute is referred to as internalized stigma [10].

COVID-19 stigma is largely based on community fear that its survivors are still contagious, therefore the present study was taken to quantify stigma attached to its survivors, and to categorize the type of stigma faced, in the light of COVID-19 pandemic.

## Methods

### Study design, setting, and participants

This was a cross-sectional, hospital-based, exploratory study conducted by the postgraduate department of psychiatry, in collaboration with the postgraduate department of chest medicine of Govt. Medical College, Srinagar. The study was conducted over one and a half months from April 15 to June 1, 2020. The survivors enrolled in our study were greater than 18 years of age and were of either gender. The study sample consisted of survivors who followed up after discharge in the outdoor patient department. The sample was selected by systematic random sampling, in which every third survivor was taken.

Survivors with a prior history of any psychiatric illnesses or chronic medical conditions like tuberculosis and human immunodeficiency virus (HIV) syndrome were excluded from the study. The participant information sheet, explaining the purpose and scope of the study was given (or read in case of illiterate) to survivors before seeking their consent to participate. They were also given an option to opt-out of the study at any point in time. The highest level of confidentiality and anonymity was maintained.

### Measures

Demographics such as age, gender, marital status, educational level, employment status, and place of residence (urban/rural), were recorded.

Stigma in this study was measured by the stigma questionnaire. The stigma questionnaire was adapted from the Ebola-related stigma questionnaire, which itself was derived from Berger’s HIV stigma scale, a validated measure of self-reported stigma in individuals infected with HIV in many cultural settings [11, 12].

The stigma questionnaire used, comprised of 15 items which measured the total stigma as enacted stigma, internalized stigma, perceived external stigma, and disclosure fears. Each item was rated on a 4-point Likert scale (0: strongly disagree, 1: disagree, 2: agree, 3: strongly agree) to describe how often the COVID-19 survivors experienced the 15 items of stigma after their discharge from the hospital. Scores were summed, with higher scores indicating greater experiences of stigma. 14 items used in our study were based on the stigma-related questionnaire used in a study done on stigma in Ebola virus disease (2014) [13]. To adapt to this disease and our setting, the Ebola-related stigma questionnaire was evaluated by an expert panel comprising of, a psychiatrist, an epidemiologist, an infectious disease specialist, and a sociologist, and was piloted in 15 COVID-19 survivors. Based on their feedback, two items from the original Ebola-related stigma questionnaire were removed; however, one new question was added [14]. The final adapted stigma questionnaire used in our study is as shown in Table 1. The reliability of the adapted stigma questionnaire was ascertained in the Kashmiri population and the Cronbach 'α' was found to be 0.92 (meaning an excellent consistency).

**Table 1:** Stigma survey items and source.

	Survey question Source(adapted from)
Enacted stigma	
1. I have been hurt by how people reacted to learning I had coronavirus disease.	Wright et. al
2. I have stopped socializing with some people because of their reactions of my having had coronavirus disease.	Wright et. al
3. I have lost friends because I had coronavirus disease.	Wright et. al
Disclosure concerns	
4. I am very careful who I tell that I had coronavirus disease.	Wright et. al
5. I worry that people who know I have had coronavirus disease will tell others	Wright et. al
Internalized stigma	
6. I feel that I am not as good as a person as others because I had coronavirus disease.	Wright et. al
7. Having had COVID-19 infection makes me feel that I am a bad person	Wright et. al
8. I feel guilty because I am COVID-19 positive	Reinius et. Al
Perceived external stigma	
9. Most people think that a person who has had coronavirus disease is disgusting.	Wright et. al
10. Most people are afraid of a person who has had coronavirus disease	The Authors
11. Most people who have had coronavirus disease are rejected when others find out.	Wright et. al
12. People I know would treat someone who has had coronavirus disease as an outcast.	Wolitski et. al
13. People I know would be uncomfortable around someone who has had coronavirus disease	Wolitski et. al
14. People I know would reject someone who has had coronavirus disease	Wolitski et. al
15. People I know would not want someone who has had coronavirus disease around their children.	Wolitski et. Al

### Data collection and ethical consideration

Responses from COVID-19 survivors were obtained by trained data collectors using self-administered or interviewer-administered (for illiterate participants) formats. All participants provided written informed consent. The consent document and the research Protocols were approved by the institutional ethics committee of Govt. Medical College, Srinagar.

### Statistical analysis

**Fig 1:**
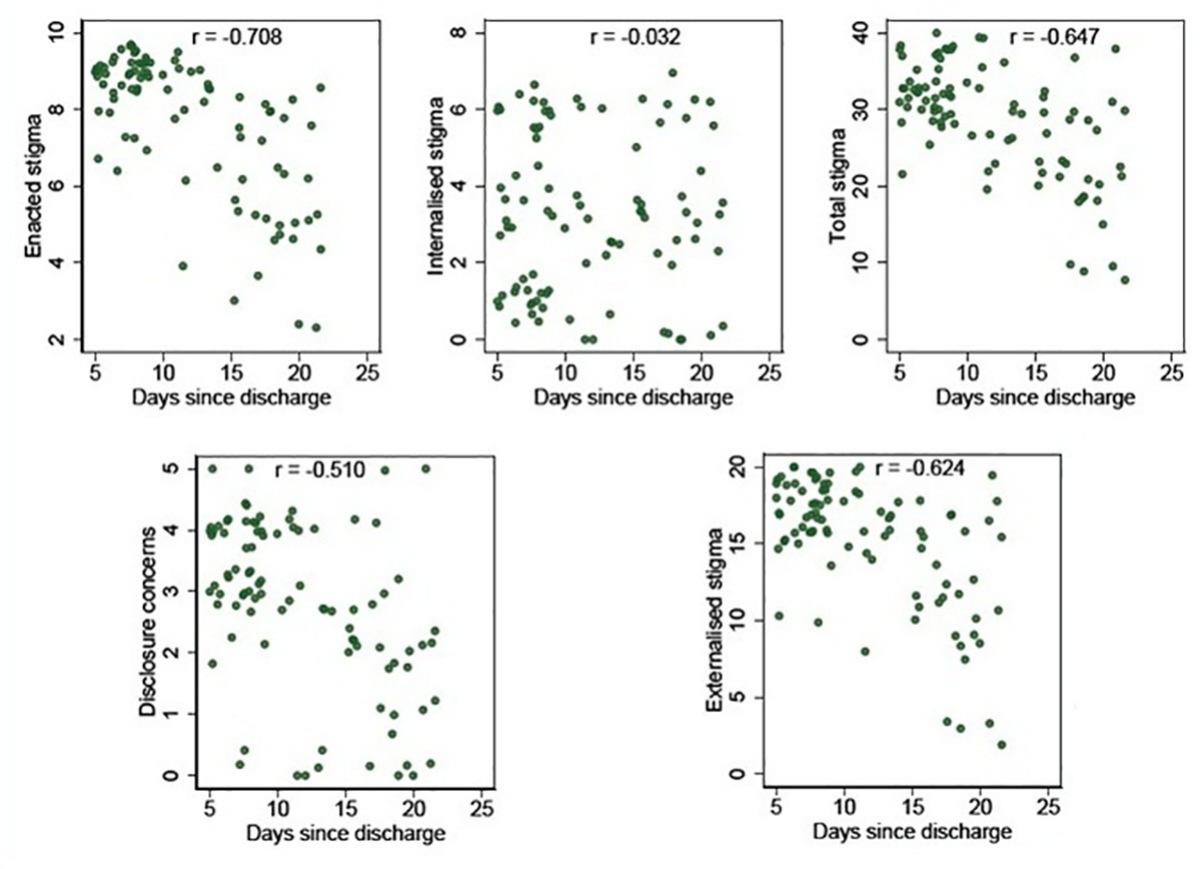
It represents the scatter plots of changes in total stigma and sub stigma scores with time since discharge.

Microsoft Excel was used for data entry. Categorical variables were summarized as frequency and percentage. Days since discharge, domain scores, and total scores were summarized as mean, standard deviation, and range. For univariable analysis with domain scores and total score as the dependent variable, the correlation coefficient was used for days since discharge, independent-samples t-test was used for dichotomous variables, and one-way analysis of variance (ANOVA) was used for categorical variables with >2 categories. For one-way ANOVA, when homogeneity of variances assumption was violated, Brown-Forsythe F was used to report the p-value. Variables found associated with the total score at p < 0.20 in the univariable analysis were used to build a multivariable linear regression model with the total score as the dependent variable; besides, the total score was also adjusted for age and gender. Two-sided p-values were reported and p < 0.05 was considered to be statistically significant. Data analysis was done using SPSS version 23 (IBM Corp. Released 2015. IBM SPSS Statistics for Windows, Version 23.0. Armonk, NY: IBM Corp.) except for Fig 1 which was made using Stata (StataCorp LLC. Stata/IC 15.1 for Windows, Revision 03 Feb 2020. College Station, TX: USA).

## Results

Out of the 95 survivors who were asked to participate in the study, four refused to consent citing health issues, as a result of which 91 survivors were taken for the final analysis. Table 2 gives a summary of the survivor’s socio-demographic characteristics. Almost half (n = 42, 46.2%) of survivors were in the age group of 30–49 years, and close to two-thirds (n = 62, 68.1%) were males, Almost three–fourths (n = 68, 74.7%) of survivors were from the urban background. The mean time from hospital discharge to study entry was 11.7±5.1 [Range(R) = 7–21] days.

**Table 2:** Participant characteristics.

		Frequency(percentages)
Age (years)	<30	30(33)
	30–49	42(46.2)
	≥50	19(20.9)
Gender	Male	62(68.1)
	Female	29(31.9)
Education	Illiterate	12(13.2)
	Studied up to 8th	15(16.5)
	Studied up to higher secondary level	28(30.8)
	Graduate	26(28.6)
	Postgraduate	10(11)
Occupation	Farmer	5(5.5)
	Businessman	21(23.1)
	Government employee	22(24.2)
	Private employee	21(23.1)
	Unemployed	22(24.2)
Marital status	Single	21(23.1)
	Married	70(76.9)
Residence	Rural	23(25.3)
	Urban	68(74.7)
Time since discharge (days)		11.7,5.1(7–21)*

*Mean, standard deviation (range)

98% of survivors provided at least one stigma endorsing response and the total mean stigma score was 28.5±7.1 (R = 6–39). The mean stigma sub-scores were 7.6±1.8 (R = 2–9) for enacted stigma, 15.0±4.1 (R = 1–20) for externalized stigma and 3.2± 2.1(R = 0–7) for internalized stigma. The mean disclosure concern subscale score was 2.7±1.4 (R = 0–5) as shown in Table 3.

**Table 3:** Type of stigma(n = 91).

	Mean(S.D)	Range
Enacted stigma	7.6 (1.8)	2–9
Disclosure concerns	2.7 (1.4)	0–5
Internalised stigma	3.2 (2.1)	0–7
Externalised stigma	15.0 (4.1)	1–20
Total stigma	28.5 (7.1)	6–39

S.D = Standard deviation

**Fig 2:**
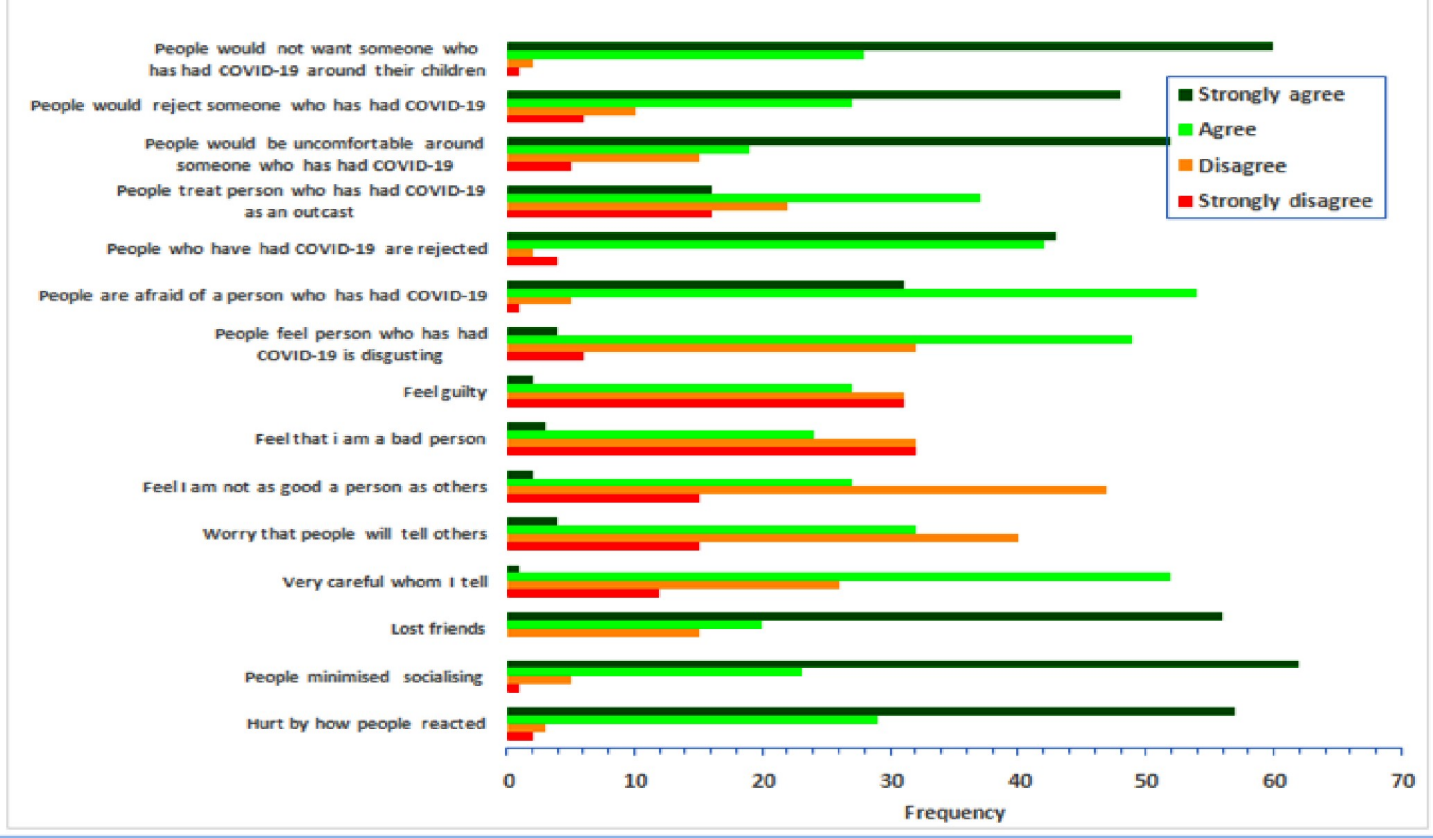
It is showing individual responses to stigma questionnaire by the study participants.

Survivors experienced the highest score in all the three questions of enacted stigma subscale (‘people minimized socializing’, ‘lost friends’, and ‘being hurt by how people reacted’). In the externalized stigma subscale survivors experienced the highest score in ‘people would not want someone who has had COVID-19 around their children’, ‘people would be uncomfortable around someone who has had COVID-19’ and ‘people who have had COVID-19 are rejected’ as can be seen in Fig 2.

Table 4 summarizes the comparison of externalized stigma, internalized stigma, enacted stigma, disclosure concerns, and total stigma, with socio-demographic variables among COVID-19 survivors. Enacted stigma was more among males as compared to females and was statistically significant (p = 0.029).

**Table 4:** Participant characteristic’s.

		Enacted stigma	Disclosure concerns	Internalised stigma	Externalised stigma	Total stigma
Age(years)	<30	7.0 (2.2)	2.6 (1.5)	3.8 (2.3)	14.2 (4.8)	27.6 (8.7)
	30–49	8.1 (1.3)	3.0 (1.2)	2.6 (1.9)	15.7 (3.7)	29.4 (5.8)
	≥50	7.5 (1.9)	2.4 (1.5)	3.4 (2.1)	14.6 (3.8)	27.9 (7.2)
	p-value*	0.070	0.264	0.055	0.290	0.565
Gender	Male	7.9 (1.6)	2.8 (1.4)	3.0 (2.1)	15.5 (4.0)	29.2 (6.8)
	Female	6.9 (2.1)	2.6 (1.4)	3.4 (2.1)	14.0 (4.1)	26.9 (7.5)
	p-value†	0.029	0.481	0.490	0.107	0.140
Education	Illiterate	6.6 (1.8)	2.7 (1.2)	4.1 (1.7)	14.2 (3.8)	27.5 (7.5)
	Studied up to 8th	7.2 (2.2)	2.7 (1.2)	3.9 (2.1)	14.1 (5.3)	27.9 (9.5)
	Studied up to higher secondary level	7.1 (1.9)	2.4 (1.7)	3.4 (2.2)	14.8 (3.8)	27.7 (6.7)
	Graduate	8.7 (0.5)	3.1 (1.1)	2.1 (1.7)	16.8 (1.7)	30.7 (2.9)
	Postgraduate	8.0 (1.6)	2.7 (1.4)	2.8 (2.5)	13.1 (6.2)	26.7 (10.7)
	p-value*	0.004	0.552	0.020	0.149	0.569
Occupation	Farmer	5.6 (1.9)	1.8 (1.3)	1.8 (2.7)	7.2 (7.4)	16.4 (13.2)
	Businessman	7.5 (1.4)	2.5 (1.7)	3.2 (2.3)	14.8 (3.3)	28.0 (6.2)
	Government employee	8.7 (0.6)	3.2 (0.9)	2.3 (1.8)	16.5 (2.6)	30.8 (3.9)
	Private employee	8.8 (0.5)	3.5 (1.0)	3.9 (2.2)	17.8 (1.4)	34.0 (3.7)
	Unemployed	5.9 (1.9)	2.0 (1.3)	3.5 (1.8)	12.7 (3.5)	24.1 (5.3)
	p-value*	<0.001	0.001	0.064	0.005	0.006
Marital Status	Single	6.9 (2.3)	2.4 (1.6)	3.3 (2.2)	13.4 (5.2)	26.0 (9.0)
	Married	7.8 (1.6)	2.8 (1.3)	3.1 (2.1)	15.5 (3.6)	29.2 (6.3)
	p-value†	0.082	0.180	0.660	0.102	0.131
Residence	Rural	7.8 (1.9)	3.0 (1.3)	3.8 (2.2)	14.7 (5.6)	29.3 (9.7)
					
	Urban	7.5 (1.8)	2.7 (1.4)	2.9 (2.1)	15.1 (3.5)	28.2 (6.1)
	p-value†	0.586	0.379	0.078	0.799	0.608

Values indicate mean (standard deviation)

* One-way ANOVA

† Independent-samples t-test

Enacted stigma and internalized stigma were both associated with education. Enacted stigma was more in highly educated in comparison to the internalized stigma which was more in less educated survivors. However, education was not significantly associated with the total stigma score (p = 0.569).Enacted stigma (p‹0.001), externalized stigma (p = 0.005), disclosure concerns (p = 0.001) and total stigma (p = 0.006) was significantly associated with occupation of the survivors. After adjusting for age, gender, marital status, and time since discharge, COVID-19 survivors who were farmers [β = -7.61, 95% CI: -12.81 to -2.42-, p = 0.005], were less likely to experience stigma as compared to unemployed. Stigma significantly decreased with increasing time since discharge after adjusting for age, gender, marital status, and occupation. [β = -0.66, 95% CI: -0.93 to -0.39, p‹0.001](See Table 5).

**Table 5:** Multivariable linear regression analysis results for total score.

Independent variables in the model		Adjusted regression coefficient (β)	Standard error	t	P value	95%confidence interval	
						Lower limit	Upper limit
Age(years)	<30	Ref					
	30–49	-0.80	1.817	-0.44	0.663	-4.41	2.82
	≥50	-0.46	1.992	-0.23	0.816	-4.43	3.50
Gender	Female	Ref					
	Male	1.36	1.320	1.03	0.304	-1.26	3.99
Occupation	Unemployed	Ref					
	Farmer	-7.61	2.612	-2.91	0.005	-12.81	-2.42
	Businessman	1.29	1.822	0.71	0.480	-2.33	4.92
	Government employee	1.22	1.966	0.62	0.535	-2.69	5.14
	Private employee	4.48	1.938	2.31	0.023	0.62	8.33
Marital Status	Single	Ref					
	Married	1.26	1.910	0.66	0.513	-2.54	5.06
Time since discharge (days)		-0.66	0.136	-4.87	<0.001	-0.93	-0.39

## Discussion

The survival of human civilization is being challenged by the emergence of COVID-19 infection, which is quickly intruding newer territories all over the globe [15]. In pandemics, there is a general increase in the stigmatization as has been seen in severe acute respiratory syndrome (SARS) epidemic or the bubonic plague [16–18]. The level of disgrace and shame linked with an infectious disease is solely based on the knowledge about the disease and the available treatment options [19]. Mass fear of COVID-19 is likely due to the uncertain character and unpredictable course of the disease, perceived risk of acquiring the infection and non- availability of FDA approved treatment, unpredictable outcome, high fatality, and novelty of the infection which can generate negative psychological responses including maladaptive behavior, and avoidance reaction among people. Thus people are likely to be labeled, stereotyped, and discriminated against, treated differently, because of real or perceived links with the disease; therefore, the first quantitative assessment study was taken to determine the prevalence of stigma, its socio-demographic correlates, and association with time since discharge among COVID-19 survivors in a developing country.

The key finding in our study is high levels of enacted and perceived externalized stigma reported by survivors. Our findings corroborate with many reports of discrimination, prejudice, and social isolation that arose during other infective pandemics [20, 21]. Our findings are in line with the findings from a comparative study in Hong Kong that reported high levels of externalized stigma in SARS survivors in comparison to HIV/AIDS, and tuberculosis [22].

Firstly stigma brings disgraces that set a person apart from others [23] and markedly increases the suffering of people with the disease. Secondly, people with the disease may hide symptoms to avoid discrimination and even conceal important travel history or those at risk of catching it may avoid seeking health care till late-thus making it easier for community transmission and harder for public health authorities to control the pandemic. Thus, such an environment can fuel harmful stereotypes and undermine social cohesion. Thirdly, Stigma can lead people to physical violence and hate crimes [24, 25]. Stigma can even make families of frontline healthcare workers pressurize them to quit jobs and de-motivate them in carrying their routine duties.

We observed relatively little internalized stigma, suggesting survivor self-worth and confidence. Our results contradict with the findings of the study on HIV/AIDS patients in Hong Kong [26] and Uganda [27], where such patients reported worthlessness, guilt, shame, and self-blame.

Our findings also partly contradict with the short term and smaller sample cross-sectional studies in Sierra Leone [28, 29], Liberia [30], Guinea [31], and DR Congo [32], which reported that EVD survivors experience several forms of internalized and enacted stigma. In contrast to our study, a high level of internalized stigma was reported in EVD survivors in Sierra Leone following their return to communities. In contradiction to our study results, A.F.Almutairi et. al found internalized stigma to be common in frontline health care workers in Middle East Respiratory Syndrome (MERS) [33].

Minimization of social communications, losing friends, being unfriended on various social media platforms, being verbally abused, being called by names, and being critically commented were reported by the COVID-19 survivors. These reports are congruent with the common forms of stigma reported by other infectious disease survivors in the literature worldwide [34]. Our findings also resonate with the previous reports of stigma in SARS victims in Hong Kong [7]. Stigma with SARS victims has been seen in many domains of everyday life, like the workplace, schools, health services, restaurants, and shopping malls. The perceived linkage between SARS and ethnicity led to the irrational avoidance of Asians (especially Chinese) in many parts of the world [35]. The stigma with SARS victims has been of such an extent that stringent restrictions were imposed on travelers from Asia [36, 37]. Many literary and media reports revealed multiple instances of survivors experiencing enacted stigma during the ongoing COVID-19 pandemic. One such report is an incident wherein a COVID-19 positive pregnant woman was abandoned, by her family after her delivery, in India [38].

COVID-19 survivors also reported that they were mocked by their communities, asked to vacate the houses by their landlords, abandoned, denied access to private transport, socially boycotted, and fired from their former private jobs [38].

COVID-19 survivors are ostracized, which prevents their social reintegration. Social isolation and losing friends can lead to increased levels of psychological distress. Disclosure concerns can lead to delayed access to medical care, low adherence to medical therapy, and reduced quality of life. Similar concerns have been reported during other infectious pandemics, as well [39, 40].

Similar results have also been reported by other qualitative studies on COVID-19, which revealed that people experienced discrimination, suspicion, and avoidance by neighborhood, insecurity regarding properties, workplace prejudice, and withdrawal from social events, even after containment of epidemics [41].

Losing jobs leaves the individuals unable to make both ends meet and this sudden misfortune adds to their guilt, frustration, depression, and mental anguish-ultimately leading to functional impairment and increased rates of suicide [42].

The common types of externalized stigma faced by COVID-19 survivors were that people would not want them around their children and people would be uncomfortable around them. This could be explained by the irrational fears of contagiousness among people about COVID-19 survivors, even after their recovery. Concordant to these findings of our study, similar concerns were reported during other infectious pandemics [13].

Our study findings reveal that enacted stigma and internalized stigma were both associated with education. Enacted stigma was more in those who were highly educated. The possible reasons for higher enacted stigma in highly educated could be that higher education is associated with better job prospects, a high chance of being employed, and more public interaction thereby more chances of encountering enacted stigma.

In our study stigma significantly decreased with increasing time since discharge. Similar results were also reported by Overholt et al who found that the levels of stigma at follow-up visits were far less than the stigma at baseline visits [13]. Thus it can be concluded that the period immediately after community re-entry is when stigmatization is at greatest. Similar results were also reported in SARS where stigma decreased, but never completely disappeared, after the outbreak [35].

The mental health impact of surviving COVID-19 is enormous, and previous studies have reported that psychological distress, anxiety, and depression are widespread among COVID-19 survivors [43, 44].

Although the impact of COVID 19 stigma on mental health of survivors is not well understood, stigma induced psychological distress and anxiety have been found to be associated with adverse mental health outcome among HIV/AIDS patients [45].

Since infectious virus diseases share similar stigmatizing attributes, it is possible that COVID-19 stigma may be contributing to the mental health complications among its survivors [46]. Thus, it is likely that stigma reduction strategies will help reduce the mental health burden among COVID-19 survivors.

### Implications

The high prevalence of enacted and externalized stigma highlights the need for consultation-liaison services to work in proximity to medical services. Apart from working in proximity to the hospital, Consultation-liaison services need to have a community-based approach as well, to ensure effective follow-up for survivors after their discharge. We hope that our study will assist and aid in planning consultation-liaison services and increase understanding of stigma associated with COVID-19 infection.

Drawing lessons from HIV/AIDS and Ebola-related stigma, several COVID-19 centered and community-driven strategies need to be framed, that could contribute to recovery and community reintegration of survivors. Long-term psychosocial community-counseling, psychoeducation, and enhancement of the coping skills of survivors should be focused upon.

Recruitment and training of trusted opinion leaders that can spread accurate de-stigmatizing messages within communities, minimizing social isolation and promoting the economic empowerment of survivors, and affected communities should be focused upon.

### Limitations

First, our study employed a cross-sectional design and, therefore we cannot infer causality between independent and outcome variables. Second, our findings are only applicable to COVID-19 survivors in Kashmir and can’t be generalized to the whole of India. Third, the majority of study participants were residents of urban environ, so survivors living in remote rural areas may have a different experience. Fourth, the period immediately after community re-entry maybe when stigmatization was greatest and maybe the over-representation of stigma. Fifth, as there is no validated measure of COVID-19 stigma, we adapted the Ebola- related stigma questionnaire, which has been validated for use in persons with Ebola virus disease.

### Future directions

1. Going forward, well-designed large scale prospective research is required to have a deeper understanding of how stigma changes over time.

2. Further research on the community reintegration experience of COVID-19 survivors is warranted.

## Conclusions

Our study results showed high levels of enacted and externalized stigma among COVID-19 survivors. Enacted stigma was more among males and in those who were highly educated. Hence there is a need to develop approaches to prevent and minimize such stigma during the ongoing outbreak and also in its immediate aftermath.

## Supporting information

S1 File (XLSX)
