# Correction: Stigma in coronavirus disease-19 survivors in Kashmir, India: A cross-sectional exploratory study

**DOI:** 10.1371/journal.pone.0244715

**Published:** 2020-12-23

**Authors:** Shabir Ahmad Dar, Syed Quibtiya Khurshid, Zaid Ahmad Wani, Aaliya Khanam, Inaamul Haq, Naveed Nazir Shah, Mir Shahnawaz, Hena Mustafa

The following information is missing from the Funding statement: The authors declare that the project was funded by National Health Mission, J&K. The funders had no role in study design, data collection and analysis, decision to publish, or preparation of the manuscript.

The Acknowledgements section for this paper is incorrect. The correct statement is: The authors are indebted to Mission director, National Health Mission, J&K for providing financial aid for this study. The authors also extend their gratitude to professor Samia Rashid, Principal/Dean Government Medical College Srinagar for her support. We also thank HOD Psychiatry, Professor Mohammad Maqbool Dar for his insightful guidance. Lastly, we thank COVID-19 survivors who participated in this study.
